# Monitoring of the Impact of Lithium Nitrate on the Alkali–aggregate Reaction Using Acoustic Emission Methods

**DOI:** 10.3390/ma12010020

**Published:** 2018-12-21

**Authors:** Justyna Zapała-Sławeta, Grzegorz Świt

**Affiliations:** Faculty of Civil Engineering and Architecture, Kielce University of Technology, Aleja Tysiąclecia Państwa, Polskiego 7, 25-314 Kielce, Poland; gswiter@gmail.com

**Keywords:** alkali–silica reaction, mitigation, lithium nitrate, acoustic emission, microstructures

## Abstract

The study analyzed the possibility of using the acoustic emission method to analyse the reaction of alkali with aggregate in the presence of lithium nitrate. Lithium nitrate is a chemical admixture used to reduce adverse effects of corrosion. The tests were carried out using mortars with reactive opal aggregate, stored under the conditions defined by ASTM C227. The acoustic activity of mortars with a corrosion inhibitor was referred to linear changes and microstructure of specimens in the initial reaction stages. The study found a low acoustic activity of mortars with lithium nitrate. Analysis of characteristic parameters of acoustic emission signals, combined with the observation of changes in the microstructure, made it possible to describe the corrosion processes. As the reaction progressed, signals with different characteristics were recorded, indicating aggregate cracking at the initial stage of the reaction, followed by cracking of the cement paste. The results, which were referred to the acoustic activity of reference mortars, confirmed that the reaction of opal aggregate with alkali was mitigated in mortars with lithium nitrate, and the applied acoustic emission method enabled the detection and monitoring of ASR progress.

## 1. Introduction

Alkali–silica reaction (ASR) is one of the reasons for damage to concrete structures [1,2]. Under high humidity conditions, aggregates that contain amorphous or poorly crystalline silica react with the alkali from the cement, forming strongly swelling reaction products. The heterogeneous nature of concrete and its low tensile strength make it susceptible to cracking due to corrosion processes. The sodium and potassium silica gel forming around the reactive aggregate absorbs water and swells, exerting pressure on the surrounding cement paste. The reaction occurs in the entire volume of concrete. Cracks of the aggregate and the matrix of the cement paste caused by the reactivity of the aggregate disrupt the integrity of concrete structure, reducing its durability [3].

The durability of concrete made of reactive aggregate or potentially reactive aggregate can be improved by using chemical admixtures, including lithium salts. The effectiveness of lithium compounds, particularly lithium nitrate, in the mitigation of damage caused by the presence of reactive aggregate in the concrete was demonstrated in the 1950s [4]. Although the mechanism of ASR inhibition by lithium nitrate has not been explained, the most frequent hypotheses refer to protection of the reactive aggregate through formation of a layer of lithium-enriched silica gels on the aggregate surface, which is impermeable to aggressive ions [5]. The latest studies conducted by Tremblay et al. [6] indicate, in turn, that lithium ions increase the stability of reactive silica, reducing its solubility for reasons other than the formation of layers of nonswelling alkali silica on the surface of reactive silica or a drop of pH of the solution in the pores.

Lithium nitrate added to the concrete mixture in a suitable molar ratio of lithium to the alkali from the cement effectively reduced the expansion of mortar/concrete to a safe level [7,8]. This ratio was in the range of 0.72 to 0.92 [9], and the amount found to be sufficient to protect most of the reactive aggregate against ASR was assumed to be approximately 0.74 [4,10,11]. Despite extensive research on the effectiveness of lithium nitrate, there are still many unclear issues, in particular, why ASR effects are mitigated in the case of some reactive aggregates while no inhibition occurs in the case of other aggregates, despite using an increased lithium nitrate dose [7,12]. It should be noted that the required amount of lithium nitrates is not correlated with the reactivity of the aggregate or its mineral composition, and determination of the correct amount of the admixture should be carried out experimentally [13].

The effectiveness of lithium nitrate in ASR mitigation is determined primarily by testing the expansion of mortars or concretes kept under conditions that accelerate the alkali–silica reaction [14,15]. In order to assess the effectiveness of a potential corrosion inhibitor, it should be indicated if it mitigates the adverse effects of the reaction, i.e. expansion, cracks, and exudations of reaction products. In actual structures, the effectiveness of applied solutions is tested by visual assessment of the material, measurements of concrete cores expansion, or petrographic and microscopic methods [16]. Fournier et al. demonstrated that measurements of length changes could be inconsistent, in particular due to temperature variations. The assessment of corrosion advancement based on an observation of cracks and exudations on the concrete surface, in turn, is frequently subjective. The petrographic or microscopic method used for a specimen cut out of the concrete core enables quick assessment of concrete damage caused by the reaction, but its accuracy and reliability depends on the skills and experience of the mineralogist. As indicated by Abdelrahman, visual and petrographic methods do not enable monitoring of corrosion processes — they can only support the identification of such processes [17]. Also, measurable expansion of the material occurs when the corrosion processes are advanced to some extent. The method of measuring linear changes does not detect material damage in the initial stages of the reaction, which is particularly important in tests of corrosion inhibitors. Introduction of methods enabling more accurate monitoring of corrosion processes, particularly nondestructive methods, could provide a useful way of assessing the advancement of ASR in concrete. 

Nondestructive methods used to examine the alkali–aggregate reaction include electric/electromagnetic, seismic, and acoustic methods. Rivard and Saint-Pierre used three nondestructive methods to assess concrete damage due to the alkali–aggregate reaction: ultrasonic wave velocities, dynamic Young’s modulus measured with resonant frequency, and electrical resistivity [18]. They pointed to the possibility of identifying zones with a loss of stiffness or compressive strength using the UPV method and measurement of dynamic Young’s modulus in laboratory specimens. However, the correlation with damage in the field was small. Lokajicek et al. stated that out of the nondestructive methods based on the development of brittle damage related to internal stresses, the acoustic emission method seemed to be the most adequate method for tests of the alkali–aggregate reaction [19]. They based this statement on a large number of tests concerning the detection of brittle fracture in rocks exposed to loads and concrete [20,21].

The acoustic emission method is noninvasive, and it is not sensitive to environmental conditions, i.e., humidity and temperature [17]. The microcracks appearing and propagating in the concrete as a result of corrosion processes generate elastic waves propagating from the source in all directions [22]. The generation of waves by active damage processes, which are recorded by a set of sensors, is used in the nondestructive, passive acoustic emission (AE) method [23,24]. The AE method enables an accurate analysis of the tested object, including detecting and locating damage, whose level of risk to the structure is identified in the form of the intensity of recorded emission signals [25]. AE was used, in particular, to analyse the extent of damage to reinforced concrete structures, by recording the formation of cracks at the reinforcement–concrete interface [26]. The cracks were caused by the formation of expansive reaction products. The effectiveness of this method was also confirmed during the monitoring of the alkali–aggregate reaction [17,19,27,28]. The acoustic emission method, however, has not been used to analyse the effectiveness of corrosion inhibitors.

The way material cracking occurs affects the characteristics of acoustic emission signals, i.e., the number of signals, signal duration, rise time, amplitude, signal energy, and frequency [29,30]. Analysis of acoustic activity parameters can be useful in determining the advancement of corrosion processes and examining the corrosion mechanism itself.

This study analyzed the possibility of using acoustic emission to assess the effectiveness of inhibition of the alkali–silica reaction using lithium nitrate. The progress of corrosion processes was tested using mortars with reactive opal aggregate, stored under the conditions defined by ASTM C227. ASTM C227 was designed to stimulate the conditions of the inside of mass concrete structure suffering from alkali–silica distress, and it is one of the methods of testing potential aggregate reactivity. Thomas et al. used the ASTM C227 method to determine the expansion of mortars with reactive rhyolite and, after adding lithium nitrate, demonstrated good correlation with the results of the expansion of mortars analyzed with the short-term method in accordance with ASTM C1260 [31]. Thus, it was demonstrated that despite certain flaws, this method could be used to assess the effectiveness of lithium compounds. The results were referred to the acoustic activity of reference mortars, which did not contain the lithium admixture. The samples were monitored continuously for 14 days in order to analyse the initial reaction stages. Additionally, the degradation of the material was observed by scanning electron microscopy coupled with energy dispersive X-ray microanalyzer (SEM/EDS), which provided a more complete description of the processes.

## 2. Materials and Methods

### 2.1. Specimens

The experiment involved two series of mortars: reference mortars and mortars with the corrosion inhibitor, 6 specimens in each series. Mortar bars with a size of 25 × 25 × 250 mm were made of CEM I 42.5R Portland cement with alkali content of 0.90 Na_2_O_e_ and natural reactive opal aggregate with grain size distribution of 0.5–1 mm. The chemical composition of the cement and petrographic composition of the opal aggregate are indicated in Table 1 and Table 2.

The reactive aggregate made up 6% of the mass of the aggregate composition; the remaining portion consisted of nonreactive silica aggregate. The aggregate-to-cement ratio was 2.25, and the w/c ratio was 0.47, as required by ASTM C227 [32]. Lithium nitrate was added with the mixing water in the amount corresponding to the molar ratio of lithium to alkali in cement, i.e., 0.74.

Mortar specimens were moved into special containers after 24 h of maturing and kept in a climate chamber under a controlled temperature of 38 ± 2 °C and relative humidity of 100%. The duration of the test was 14 days.

### 2.2. Expansion Measurements

Linear changes of reference mortars with the lithium nitrate were measured throughout the experiment, in reference to the baseline measurement, i.e., reading of specimen dimensions after 24 h of bar storage in the climate chamber. The specimens were measured using the Graff–Kaufmann (produced by The Institute of Ceramics and Building Materials, Division in Opole, Poland) apparatus with an accuracy of down to one 0.005 mm. The measurements were conducted each day, immediately after the bars were removed from the containers. 

### 2.3. Acoustic Emission

**Figure 1:**
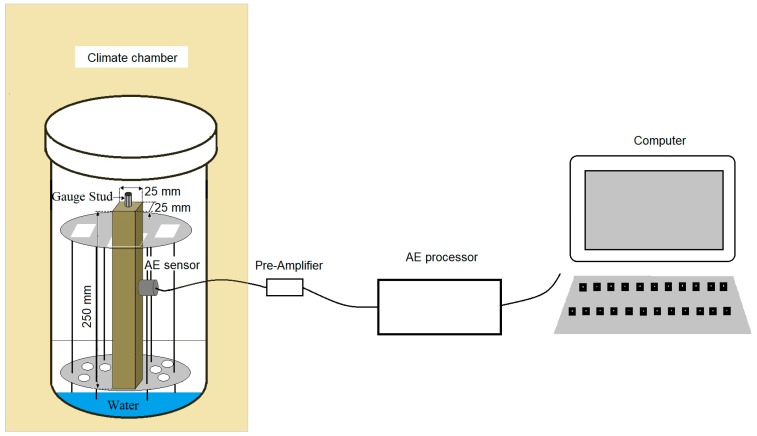
Schematic diagram for a single specimen of acoustic emission (AE) signal processing.

Three specimens of mortars in each series were monitored using the AE method. The mortars were stored under the conditions defined in ASTM C227. Mortar bars were placed vertically in racks inside sealed containers under controlled temperature and humidity conditions. The specimens were placed above the water surface. Three specimens of each series were placed in each container. Every specimen was connected to its measurement channel. A resonant piezoelectric sensor with a frequency of 55 Hz was attached to each specimen with a tape and connected to the surface of the mortar using a silicone sealant (Figure 1). Sensor calibration was conducted before the actual measurements by reading the parameters of AE signals generated by a reference source. The reference signal was the HSU-NILSEN source—acoustic signal generated by the fracture of the 2H graphite lead of a pencil on the surface of the mortar. The sensor system was connected to a 24-channel µSAMOS processor. The signals were recorded continuously for 14 days.

The effectiveness of acoustic emission tests strongly depends on the procedures for digital processing of signals in order to reduce background noise [33]. In order to limit the recording of so-called noise, suitable background noise filters were used: HDT (hit definition time), determining the time between the end of one signal and beginning of another signal; HLT (hit lockout time) to prevent measurement of reflected signals; and PDT (peak definition time), set as, respectively, 800 µs, 1000 µs, and 200 µs. The specified 40-dB noise threshold for all sensors enabled the elimination of background signals, e.g., operation of the chamber.

### 2.4. Microstructure of Polished Section

Strips with a size of 25x25x10 mm were cut out of the mortar specimens after 1, 3, 7, and 14 days of reaction. The specimen surface was ground and then polished on a diamond washer. The microstructure of the mortars was observed in the backscattered electron (BSE) detection system under the scanning electron microscope (SEM; Quanta FEG 205, FEI Company, Hillsboro, OR, USA). Images of the microstructures were produced with accelerating beam voltage of 15 kV.

## 3. Results

### 3.1. Expansion

**Figure 2:**
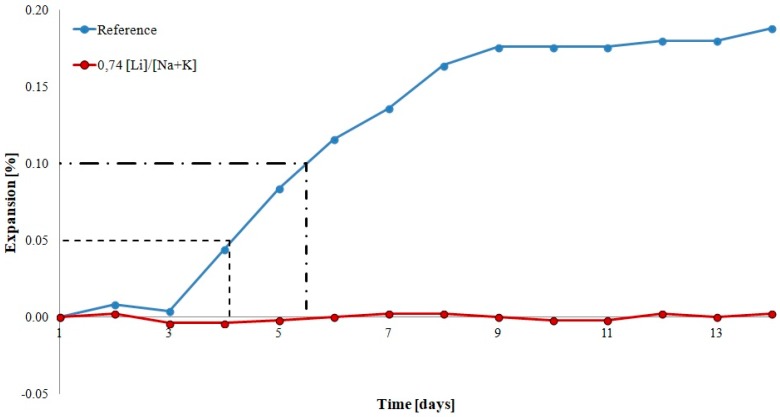
Expansion of mortars with reactive opal aggregate.

Figure 2 shows the expansion of mortars with opal aggregate with and without lithium nitrate. Dashed lines were used to mark the thresholds of potential aggregate reactivity. In accordance with ASTM C227 and supplementary annex C33 to the standard, expansion of mortars exceeding 0.05% by the 90th day of reaction and 0.10% after 90 days is a reason to classify the particular aggregate as reactive. In the analyzed case, the expansion of reference mortars exceeded 0.05% after the 4th day and 0.10% after the 5th day of reaction, indicating that the analyzed aggregate was potentially reactive and reacted quickly. In the case of mortars with lithium nitrate, expansion was reduced to a safe level, not exceeding 0.05% in the analyzed period.

**Figure 3:**
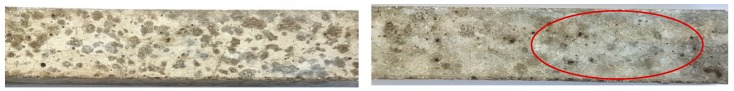
Exudations on the mortar surface (**a**) reference and (**b**) with lithium nitrate.

Observation of the surface of the bars indicated a significant progress of the reaction in the reference mortars, manifesting in the form of multiple exudations. Exudations of reaction products were also observed on the surface of mortars with lithium nitrate, but to a much smaller extent than in the specimens without the admixture (Figure 3). Although the mortars with lithium nitrate did not demonstrate expansion, opal aggregate reacted with alkali, but to a smaller extent than in the mortars without the corrosion inhibitor.

### 3.2. Acoustic Emission of Mortars

**Figure 4:**
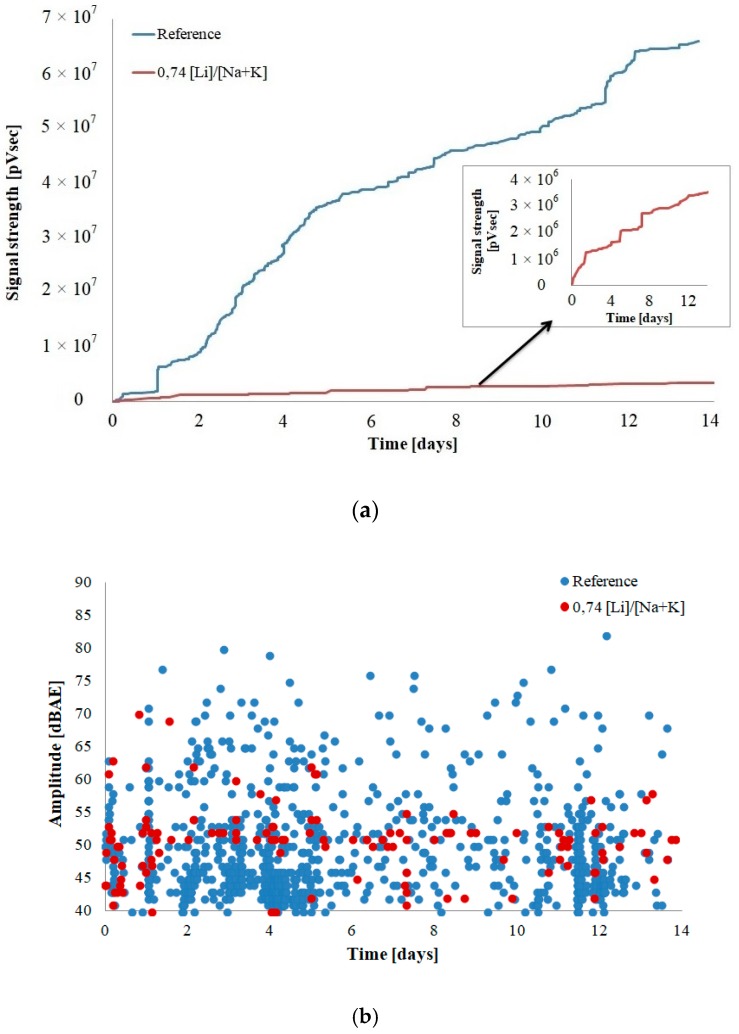
AE characteristics of mortar bars (**a**) signal strength and (**b**) amplitude.

**Figure 5:**
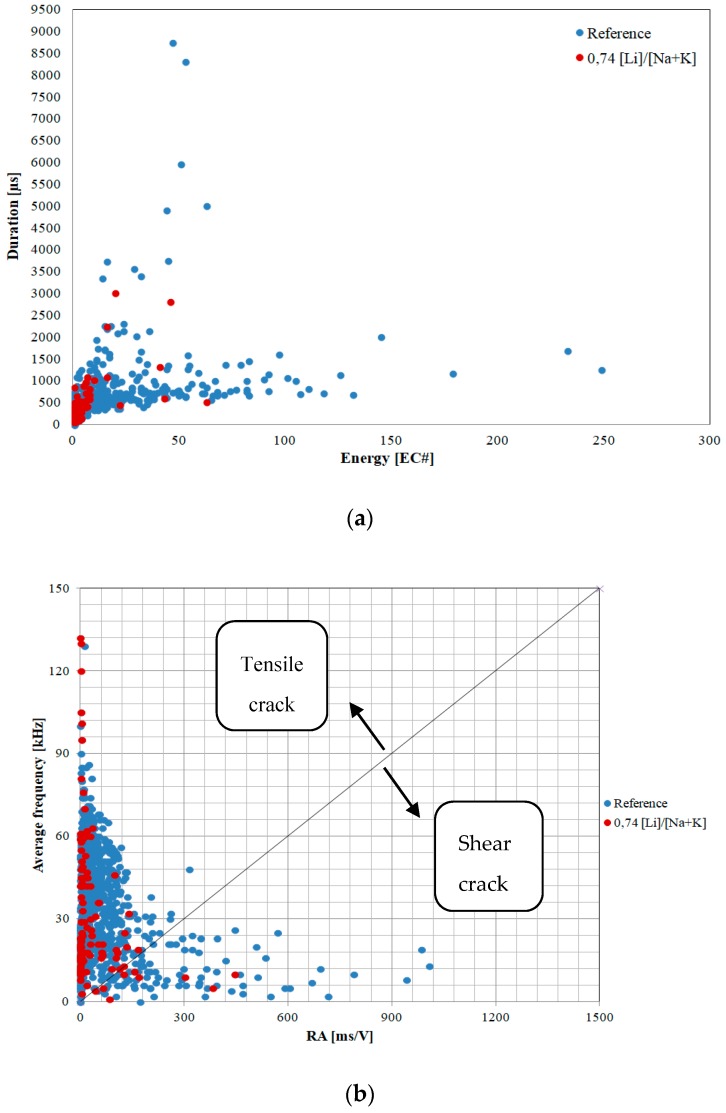
Duration versus energy (**a**) and average frequency versus RA index (**b**) for AE events of mortar samples.

Figure 4 and Figure 5 depict filtered results of acoustic activity of the control mortar and the mortar with lithium nitrate in the initial 14-day period of alkali–silica reaction.

The strength of the signals recorded in the mortars with the corrosion inhibitor is approximately 20 times smaller than the strength of signals emitted by the reference mortars (Figure 4a). With the progress of the experiment, the strength of the signals increases, without flattening out, similarly to the reference mortars, throughout the analyzed period. The cumulative curve of signal strength indicates a rapid increase on the 2nd, 5th, and 8th day of the experiment. The values of signal amplitude in the mortars with lithium nitrate are lower as well and do not exceed 70 dBAE. The phenomena recorded in the mortars with the inhibitor are primarily mild, indicating gradual cracking in the material structure, as opposed to the large and quick activity of the reference mortars (Figure 4b).

The duration and energy of the recorded signals were analyzed in order to determine the advancement of corrosion processes (Figure 5).

AE signal duration is a time (in μs) during which the amplitude of the signal exceeds the threshold value. Signal duration is a parameter frequently used in damage analysis. Signal energy is an area limited by the envelope of extreme values of the AE signal, measured in 1 μVs/count. Both the duration and energy of acoustic emission signals recorded in the mortars with lithium nitrate were lower than the ones recorded in the mortars without the admixture. The recorded signals had energy of up to 63 EC# and duration of approximately 3000 µs, whereas in the reference mortars, the energy was 250 EC# and the duration of the longest signal exceeded 8500 µs. The values of these parameters are indicative of the formation and propagation of microcracks also when the corrosion inhibitor is present, but to a much smaller extent than in the reference mortars. Aggregate cracks are caused by swelling alkali gels produced by the alkali–silica reaction. Signals with similar parameters were recorded in concrete structures with cracks visible on the surface. The visual analysis of the specimens did not demonstrate visible cracks on the surface of the mortars, in both the reference mortars and the mortars with the corrosion inhibitor.

Information about the source of acoustic emission signals can be obtained from the dependence of the average frequency (AF) and the RA factor, i.e., the product of signal rise time and maximum amplitude [34]. RA and AF factors may be useful in classifying the cracks by indicating cracks caused by tension and shear. The factors have been determined in accordance with Formulas (1) and (2):

(1) $$RA=\frac{RT}{A}$$

(2) $$AF=\;\frac{C}{D}$$

where, RT—rise time

A—peak amplitude

C—total number of threshold crossings for an AE waveform.

D—duration of an AE waveform

These parameters were used to assess the condition of reinforced concrete members. The straight line separating the area of the chart into two zones can be used to indicate the source of cracks caused by tension or shear. The slope parameters of the straight line determined using the Japanese JCMS-IIIB 5706 (2003) [35] standard was 0.1 Hz*s/V, but different values are also sometimes adopted in professional literature [21]. Signals with parameters higher than 0.1 Hz*s/V are classified as caused by tensile stresses, and signals with lower values are classified as shear cracks. When the signals are close to 0.1 Hz*s/V, the cracks are a combination of shear forces and tensile forces [36]. Signals emitted by mortars with the corrosion inhibitor have an average frequency comparable to the reference mortars. Significant differences, however, were observed in the values of the RA factor. The results suggest that the source of acoustic emission in the mortars with lithium nitrate was primarily the cracks of the reactive aggregate. Signals indicating the action of shear forces indicate cracks primarily at the interface between paste and aggregate. The number of such cracks is much smaller than in the mortars without the corrosion inhibitor.

### 3.3. Microstructure of the Mortars

Observations of the microstructure of the mortars, carried out in parallel to the measurements of acoustic emission, enabled a fuller interpretation of the results. It should be noted that the number of corrosion spots throughout the analyzed period of the experiment in the mortars with the corrosion inhibitor was much smaller than in the mortars without the lithium admixture.

**Figure 6:**
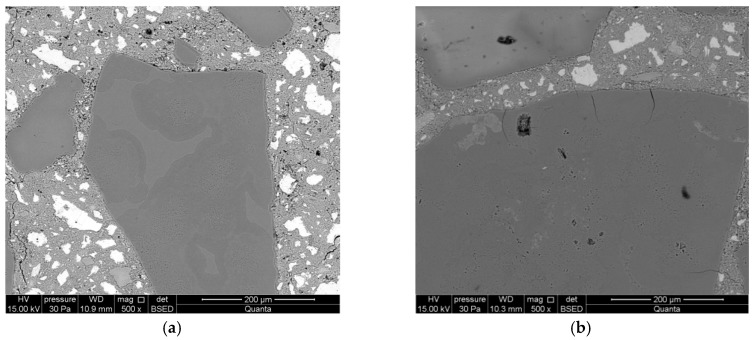

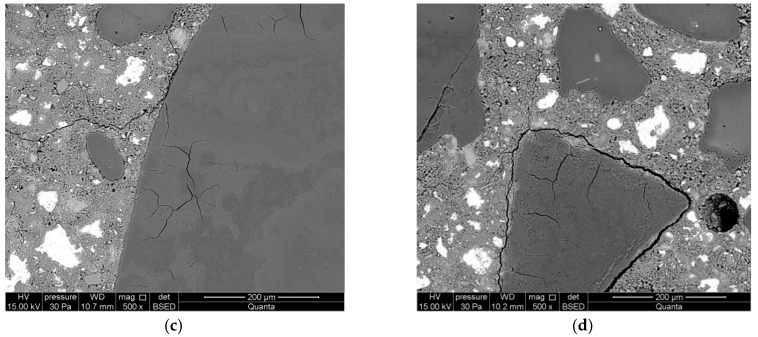
Microstructure of mortars with lithium nitrate, analyzed along with the progress of the reaction after: (**a**) 1 day; (**b**) 3 days; (**c**) 7 days; and (**d**) 14 days.

On the first day of reaction, partial dissolution of reactive aggregate and formation of alkali silica gel in their place were observed. No cracks were found in the aggregate (Figure 6a).

Longer exposure of mortars to conditions that accelerate the corrosion processes manifests in further dissolution of reactive silica and aggregate surface cracks. The forming gels demonstrate stronger swelling properties than the gels formed after 1 day (Figure 6b). A significant intensification of alkali–silica reactions was observed after 7 days of reaction. Aggregate cracks were observed both on the surface and inside the aggregate (Figure 6c). Debonding of reactive aggregate from the cement matrix was found during further reaction, after 14 days of exposure (Figure 6d). Smaller cracks in the paste propagate from the crack forming along the aggregate boundary. A massive alkali–silica gel has formed on the aggregate surface.

**Figure 7:**
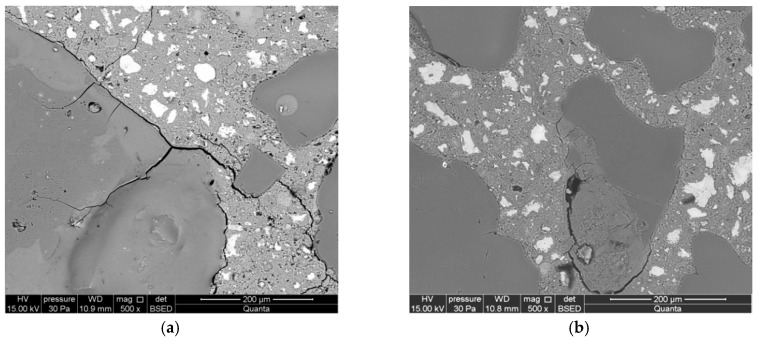

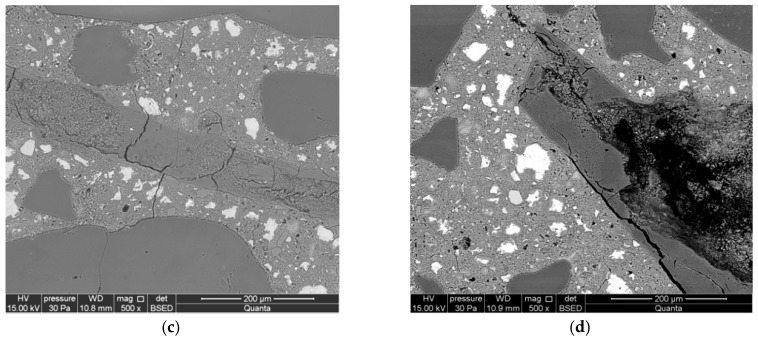
Microstructure of reference mortars, analyzed along with the progress of the reaction after (**a**) 1 day, (**b**) 3 days, (**c**) 7 days, and (**d**) 14 days.

The development of corrosion processes in the reference mortars is different. The opal aggregate was considerably damaged already during the first day of reaction (Figure 7a). Cracks were found both in the aggregate and in areas from the aggregate towards the cement matrix. During later stages, the reaction products occupied the area of reactive aggregate, also filling the cracks formed in the aggregate (Figure 7b). The aggregate was partially debonded from the cement matrix.

Cracks passing through the aggregate were observed in the reactive aggregate after seven days of reaction. The advancement of the reaction was much greater in smaller opal aggregate. The cracks are propagating from the reactive aggregate to the matrix of the cement paste. Reaction products have been identified inside the aggregate (Figure 7c). The microstructure of mortars after 14 days is characterized by an increased size of cracks in the aggregate and a significant degree of reacted large-sized aggregate (Figure 7d).

## 4. Discussion

This study presented the possibility of using acoustic emission to test the effectiveness of inhibition of the alkali–silica reaction. The acoustic activity of the reference mortars was compared to the activity of mortars containing lithium nitrate. The test results were referred to measurements of expansion of mortars and the image of their microstructure.

Large acoustic activity of specimens with reactive opal aggregate was due to the formation and propagation of microcracks in the aggregate, at the interface between aggregate and cement matrix and in the matrix itself. The damage was caused by the calcium-alkali–silicate hydrate gel formed as a result of the alkali–silica reaction, which demonstrated swelling properties. In the presence of lithium nitrate, material damage is mitigated due to the reduced corrosivity of reactive aggregate. Mortars with lithium nitrate demonstrate small acoustic activity, which confirms the effectiveness of the admixture as a corrosion inhibitor. Corrosion processes take place, but to a much smaller extent than in reference mortars. The recorded signals had small strength and even smaller amplitude, indicating less material damage. The reduction of cracks in mortars with lithium nitrate was confirmed by the parameters of the recorded signals, i.e., signal energy and duration. Smaller energy and shorter duration of the signals in comparison with the signals recorded in the mortars without the admixture indicate that the reaction products do not generate as much swelling pressure as the products formed in the reference mortars.

The degree of the acoustic activity of the specimens was referred to the measurements of their expansion. No expansion of mortars was recorded after the introduction of lithium nitrate. A small shrinkage is caused by the imperfection of the testing method, which depends on specimen storage conditions.

The acoustic activity of the mortars with lithium nitrate is confirmed by microstructure observations. The reactive aggregate is dissolved, and swelling reaction products are formed in its place, causing aggregate cracking and debonding from the cement matrix. No protective product layer was found at the interface between aggregate and cement paste. Throughout the analyzed period, the opal aggregate was subject to damage processes, but in the specimen with lithium nitrate only a minor amount of alkali ASR gel was detected.

Results of tests of the acoustic activity of mortars with lithium nitrate are well-correlated with the results obtained by scanning electron microscopy. The analysis of the dependence of the RA factor and the average frequency of the signals demonstrated that microcracks in the mortars with lithium nitrate were formed primarily in the aggregate, with little propagation towards the cement matrix. The images of the microstructure confirmed the formation of an expansive silica–alkali gel, causing cracking of the aggregate and the interface zones between the reactive aggregates and the paste. No increase of specimen expansion (determined by linear measurements) was observed during the same period. This proves that the acoustic emission method is more sensitive, which seems particularly important during the assessment of the advancement of corrosion processes, particularly in early reaction stages.

## 5. Conclusions

Based on the analyses, the following has been found.

- The acoustic emission can be used as a tool for continuous monitoring of phenomena occurring during the alkali–aggregate reaction (SHM method).
- The AE method could be used to assess the effectiveness of inhibitors of the alkali–aggregate reaction. The reduction of degradation in concrete with reactive aggregates caused by the use of lithium nitrate was confirmed by the reduced acoustic activity.
- Analysis of energy parameters of acoustic emission signals, i.e., signal strength, amplitude, and duration, can be used to determine the intensity of corrosion processes.
- The acoustic activity of the mortars is well-correlated with the extent of damage to their microstructure.
- Analysis of RA and AF parameters can be used to identify the type of stresses that cause damage to concrete microstructure. In mortars with lithium nitrate, the cracks, which are recorded primarily in reactive aggregate, do not propagate to the cement paste to the same large extent as in the mortars without the admixture. This indicates smaller advancement of corrosion processes. Analysis of RA and AF factors, therefore, may be useful in determining the extent of concrete degradation due to the alkali–aggregate reaction.

Further research should be carried out on the use of pattern recognition and neural networks to select and create a set of reference signals describing these phenomena.

## Figures and Tables

**Table 1:** Chemical composition of cement.

Material	SiO_2_	Al_2_O_3_	Fe_2_O_3_	CaO	MgO	SO_3_	K_2_O	Na_2_O	P_2_O_5_	LOI ^a^	N.s.p ^b^
Cement	19.07	5.43	2.79	62.99	1.66	3.41	0.99	0.25	0.45	2.25	1.00

^a^ LOI — loss of ignition. ^b^ N.s.p — parts nonsoluble in HCl and Na_2_CO_3_.

**Table 2:** Opal aggregate composition.

Constituent	Opal	Chalcedony	Quartz	Talc	Goethite	Pores	Sum
Content [%vol.]	65	30	2	1.5	1	0.5	100
